# Isolated Bilateral Triphalangeal Thumb with Delta Phalanx: A Case Report

**Published:** 2013-06

**Authors:** Jamal Gousheh, Ehsan Arasteh

**Affiliations:** Department of Plastic and Reconstructive Surgery, Shahid Beheshti University of Medical Sciences, Sheikh Bahai Medical Center, Tehran, Iran

**Keywords:** Triphalangeal thumb, Delta phalanx, Wedge osteotomy, Arthrodesis

## Abstract

Triphalangeal thumb is characterized by the interposition of an extra-phalanx between two normal ones. In this article the authors present the case of a 24-year-old man with bilateral triphalangeal thumb of opposable type, without any other associated anomaly or genetic syndrome. The patient had triangular delta extra-phalanxes that caused ulnar deviation of both thumbs. Surgical procedure for the correction of the congenital anomaly consisted of a closing wedge osteotomy and distal interphlangeal joint arthrodesis in the left thumb, and a wedge osteotomy in the deformed distal phalanx of the right thumb. Appearance and precision function of hands considerably improved 6 months after the operation, and there was no major stiffness in proximal interphalangeal joints of thumbs.

## INTRODUCTION

Triphalangeal thumb is a relatively rare congenital anomaly that is characterized by the interposition of an extra-phalanx between two normal phalanges of the thumb. Adults with triphalangeal thumbs insist that their thumbs are normal and that they can perform any task they need with the affected hands. However, the thumb often appears grossly deformed, and observation of functional movements reveals that the extra length and deviation significantly alters the thumb’s functional precision. The etiology of the condition has been linked to inheritable traits and teratogenic agents such as thalidomide.^1^^-^^3^

Triphalangeal thumb occurs as an isolated defect, in association with other malformations of the hand, or as a feature of malformation syndromes. This anomaly can be subdivided into opposable and non-opposable types and has been reported to occur bilaterally in 40% to 80% of cases.^4^^-^^7^

Triphalangeal thumb can be classified based on the shape of the extra-phalanx into three types of full, rectangular, and delta.^5^ The shape of extra phalanx determines whether the thumb will ultimately deviates laterally, or be too long. For the isolated anomaly, the first aim of surgical treatment is mainly aesthetic to improve the long bending appearance.^5^

Of course, it will usually improve functional abilities in precision work as well. In this article, the authors present a case of an adult man with isolated bilateral triphalangeal thumb with delta extra phalanxes.

## CASE REPORT

A 24-year-old shopkeeper male patient was referred with deviated and malformed thumbs of both hands for the correction of appearance of thumbs. Otherwise, he had a good general health, and no congenital heart disease, anemia, genetic syndromes, or other concomitant disorders in hands or feet. No similar case was reported in his family history as far as it could be traced.

**Fig. 1:**
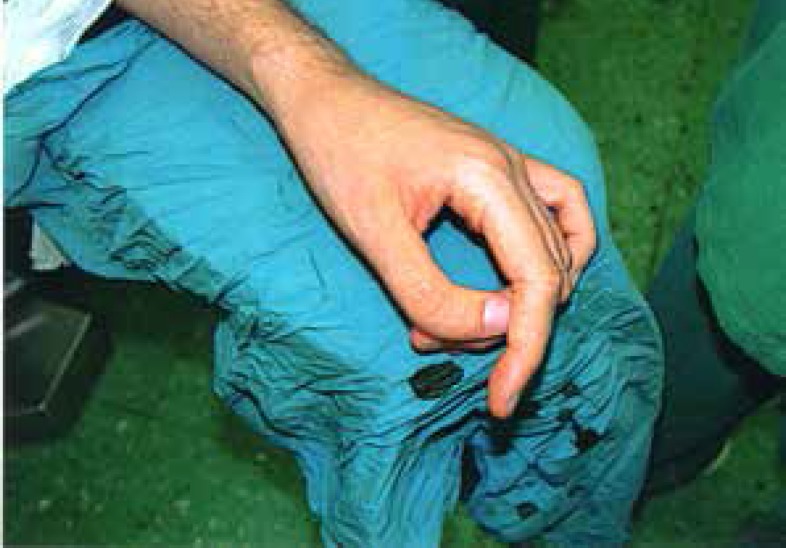
Left hand before operation. The thumb deviation is about 40 degrees

**Fig. 2:**
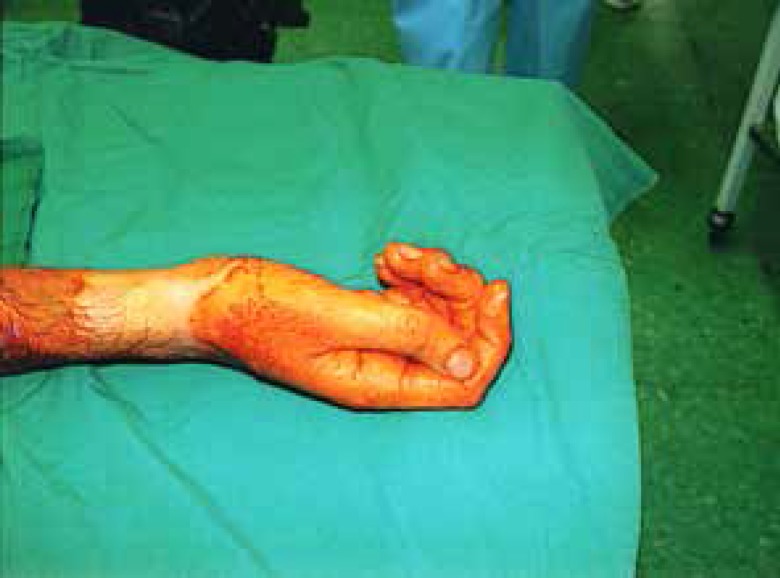
Right hand before operation. The thumb deviation is about 25 degrees

**Fig. 3:**
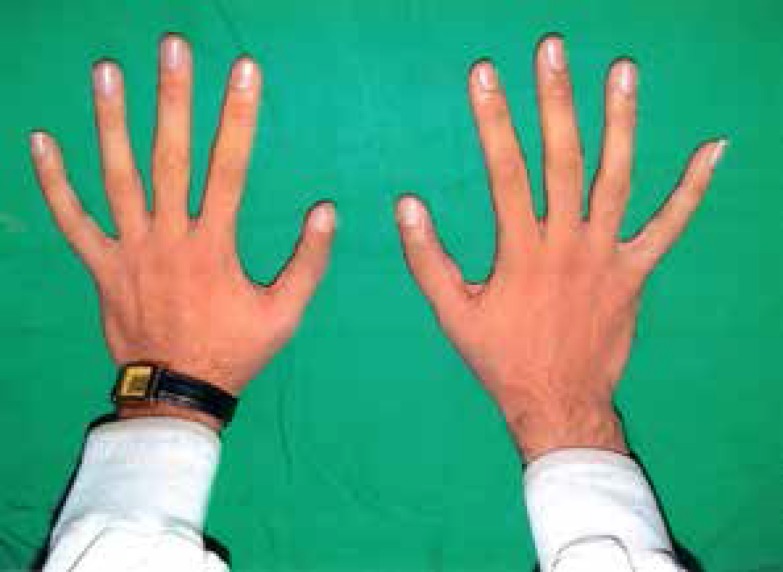
Hands 6 months after the operation. No significant deviation in thumbs remains

He was able to oppose his thumbs, and first webs and thenar muscles were normal. The only positive finding in the physical examination was a 40 degree ulnar deviation in the left thumb (Figure 1), and a 25 degree deviation in the right thumb (Figure 2). X-rays revealed a triangular delta extra-phalanx in left, and a minute similar extra-bone in right thumb, which caused ulnar deviation of distal phalanxes (Figure 3).

The patient underwent operation for the correction of both thumbs. In the left hand, through an incision in radial side of the thumb, we performed a closing wedge osteotomy, articular cartilage from both sides of the DIP joint were removed, followed by fusion with K-wire. PIP joint was left undisturbed. In the right hand, extra phalanx was very small, and resembled a sesamoid bone. 

**Fig. 4:**
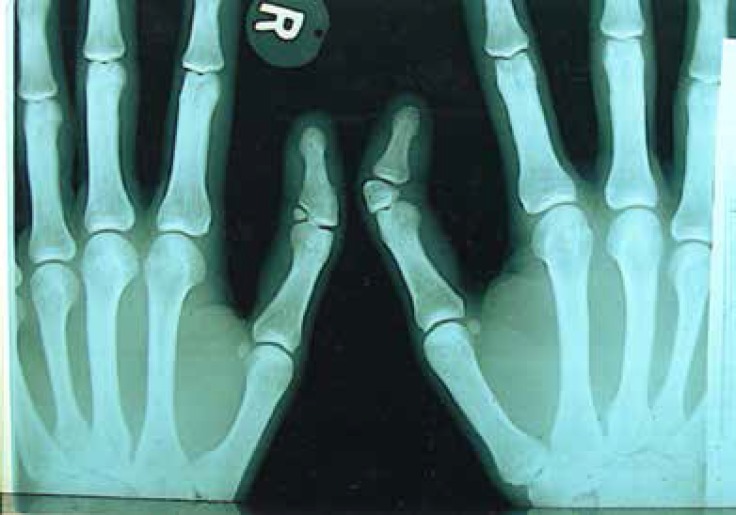
X-ray views of both thumbs. Delta extra-phalanxes and deviation before the operation are displayed

We performed a closing wedge osteotomy at the base of distal phalanx, in its radial side, and fixed it with a K-wire. Six week post-operatively, K-wires were removed. Healing process ended without any complication. After a follow up period of 6 months, the patient was satisfied with the new appearance of his thumbs. In the physical and X-ray examinations deviation in the thumbs was improved significantly to less than 10 degrees (Figure 4). 

**Fig. 5:**
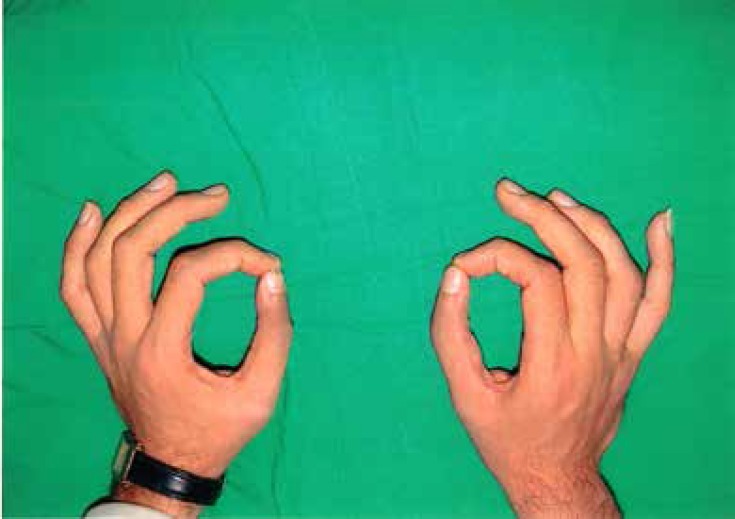
Pinching of both thumbs has improved 6 months after the operation

An improvement in gripping and precise pinching, and perfect thumb opposition was observable post-operatively (Figure 5). No major late postoperative complication was recorded. The un-touched PIP joint was minimally stiffed and maximum flexion in IP joints of thumbs were 40 degrees. No tenderness or articular instability was detectable.

## DISCUSSION

Although the mere presence of an abnormally shaped phalanx should not be considered an indication for operative treatment, most adults find their long and deviated thumb a handicap with precision work. Surgical intervention for these patients may be considered to correct thumb angulation and length, and stabilization the joint. Contrary to this patient, other associated abnormalities, such as polydactyly, web contracture, and thenar muscle atrophy may complicate the situation and necessitate additional operative procedures.^2^

Since the end of 19^th^ century, several surgical modalities have been explained for treatment of triphalangeal thumb. Removal of accessory phalanx^8^^-^^10^ as an operative option is technically simple. However, this procedure may result in an aesthetically unpleasing or unstable thumb. Furthermore, reconstruction of collateral ligaments is difficult or nearly impossible in adult patients.

Another alternative is to perform reduction osteotomy. The extrinsic flexor and extensor tendons may also shorten.^11^^,^^12^ In the rare instance when an older child or adult has a deviated thumb and a stiff DIP joint, the delta phalanx is sometimes best left undisturbed and alignment is achieved by a closing wedge osteotomy at the level of proximal phalanx shaft.^2^

For the case reported in this article, a closing wedge osteotomy and arthrodesis was performed. Therefore reconstruction of the joint capsule and ligaments was not required. The end results were excellent. Deviation and instability of thumbs were relieved, and PIP joint did not develop major stiffness as a complication of surgery. Finally the patient was satisfied with the new appearance of his hands and his ability with precision work was improved.

## CONFLICT OF INTEREST

The authors declare no conflict of interest.

## Footenote

Please cite this paper as:

Gousheh J, Arasteh E. Isolated Bilateral Triphalangeal Thumb with Delta Phalanx: A Case Report. World J Plast Surg 2013;2(2): 111-114.
